# Design of 3D printing osteotomy block for foot based on triply periodic minimal surface

**DOI:** 10.1038/s41598-024-65318-4

**Published:** 2024-07-09

**Authors:** Hai-qiong Xie, Hai-tao Xie, Tao Luo, Bai-yin Yang, Dao-qi Gan, Dong-fa Liao, Lin Cui, Lei Song, Mei-ming Xie

**Affiliations:** 1 School of Advanced Manufacturing Engineering, Chongqing University of Posts and Telecommunications, Chongqing, 400065 People’s Republic of China; 2 XingGuo People’s Hospital, Jiangxi, 341000 People’s Republic of China; 3 Trauma Center, General Hospital of Western Theater Command of PLA, Rongdu Str. 270, Chengdu, 610083 People’s Republic of China; 4 Department of Orthopaedics, First Affliated Hospital, Army Medical University, No. 30 Gaotanyanzheng Street, Chongqing, 400038 People’s Republic of China

**Keywords:** Triply periodic minimal surface, Three-dimensional printing, Osteotomy, Foot, Anatomy, Medical research

## Abstract

The ankle joint, which connects the lower limbs and the sole of the foot, is prone to sprain during walking and sports, which leads to ankle arthritis. Supratroleolar osteotomy is an ankle preserving operation for the treatment of ankle arthritis, in which the osteotomy is an important fixing and supporting part. In order to avoid stress shielding effect as much as possible, the osteotomy block is designed as a porous structure. In this study, the osteotomy block was designed based on three-period minimal surface, and the designed structure was manufactured by 3D printing. The mechanical properties of different structures were studied by mechanical test and finite element simulation. In mechanical tests, the Gyroid structure showed a progressive failure mechanism from bottom to bottom, while the Diamond structure showed a shear failure zone at 45° Angle, which was not conducive to energy absorption and was more prone to brittle fracture than the Gyroid structure. Therefore, the Gyroid structure is valuable for further research in the development of porous osteotomy.

## Introduction

As a key load-bearing joint, the ankle joint is composed of the medial malleolus, lateral malleolus and tibiotalar joint. The main function is to stabilize load bearing and flexible movement, with a total area of about 12 cm^2^, of which the largest area is the tibiotalar joint, about 7 cm^2^. The stability of the foot is mainly provided by soft tissues such as bone tissue, ligaments and muscles near the ankle joint. Ankle arthritis is a common and frequently occurring disease, which is mainly divided into valgus type and varus type ankle arthritis. About 70% of ankle arthritis is accompanied by varus deformity^1^. Following the destruction of the foot bone and joint, surgical intervention in the form of either joint fusion or replacement becomes imperative^2–4^. The autogenous bone grafting procedure is associated with complications, including significant trauma and pain experienced at the donor site^5^. To tackle these challenges, osteotomy blocks play a pivotal role in offering structural support and serving as substitutes for damaged bones during ankle surgery. The main function of suprapolar osteotomy is to readjust the ankle joint force line, delay the ankle joint progression, restore part or even all the ankle joint function, and relieve the symptoms of patients. CHOI et al.^6^, in order to explore the influence of medial open supralkal osteotomy combined with fibula osteotomy on the pressure of the tibial talus joint and talofibular joint, selected three different tibial osteotomy Spaces (6 mm, 8 mm, 10 mm) in 10 cadaver models, and measured the pressure of the tibial talus joint and talofibular joint before and after the fibula osteotomy. The results showed that, Pfibular osteotomy can reduce the pressure on the talofibular joint without affecting the contact pressure and peak pressure of the tibiotalar joint, so PFIbular osteotomy may be necessary, especially if the osteotomy space is relatively large. In a study comparing the treatment of consistent and non-consistent medial ankle arthritis, Suh et al.^7^ found that Supracleolar osteotomy with fibula preserved was effective in the treatment of early to middle medial ankle arthritis. Osteotomy block implants traditionally used for osteotomy are generally autologous bone or allogeneic bone, but the use of human bone as osteotomy block implants may lead to rejection, disease transmission and high risk of complications^8,9^. With the development of medical additive manufacturing, porous osteotomy implants are considered an attractive alternative to autologous or allogeneic bone.

3D printing is considered a better choice of fabrication in almost every industry due to its unlimited design freedom, optimization, light-weight and customization^10^. The progress made in 3D printing technology has rendered it viable to integrate porosity within solid bone graft implants^11,12^. Currently, the commonly used medical porous metal implants are generally prepared by two 3D printing technologies, Selective Laser Melting and EBM. In order to make the surface of the implant rough and provide a better environment for adhesion and reproduction of cells and bone tissue, EBM became the manufacturing method used in this study. A rough surface can also provide a greater friction coefficient to reduce the risk of sliding out of the porous osteotomy implant.

Owing to the considerably elevated elastic modulus exhibited by conventional solid metal bone graft implants in comparison with that of human bone tissue, stress shielding may readily ensue. The incorporation of a porous structure in implant design not only enables the development of lighter weight implants, but also facilitates enhanced space for bone tissue cell growth on both sides of the osteotomy plane. Furthermore, it effectively reduces the elastic modulus of the bone graft implant and minimizes the occurrence of stress shielding^13^.

Therefore, the use of bone scaffolds characterized by bioinspired structures has received great attention^14^. Three-period minimum surface (TPMS) is a geometric structure with the advantages of full connectivity, high specific strength, high stiffness and zero mean curvature. TPMS is widely found in insect wings and animal exoskeletons, and has similar structural characteristics to trabeculae^15^. Therefore, researchers incorporated TPMS into the porous design of bone scaffolds. In recent years, TPMS have garnered significant attention from researchers in the field of 3D printing porous structures due to their unique properties. TPMS surfaces exhibit exceptional smoothness and interconnected pore heights, enabling precise control over the overall structure through implicit functions^16^. Consequently, it offers an exceptional solution for the design and modeling of porous structures in 3D-printing bone grafts^17–19^.

In this study, the porous structure was designed based on TPMS and manufactured using 3D printing technology. Mechanical tests were conducted on the samples to compare the mechanical properties of different structures. Based on this, the optimized design of the osteotomy block and the finite element analysis were carried out.

## Materials and methods

This study was approved by the Medical Ethics Committee of The General Hospital of Western Theater Command (20190062). All methods were performed in accordance with the relevant guidelines and regulations.

### Design and manufacturing

According to the form and characteristics of human bone tissue, the porosity, elastic modulus and specific surface area of the porous structure are mainly considered when designing the porous structure. Porosity is an important parameter affecting the mechanics and permeability of porous bone. Elastic modulus is an important index to evaluate the mechanical properties of porous structures and a key factor to maintain the balance between porous osteotomy implants and bone tissue. The specific surface area is the ratio of the total surface area to the total volume of the porous structure. The larger the specific surface area, the stronger the adsorption capacity, and the larger the contact area for cell proliferation, migration and attachment^20^. The design of porous unit structures utilizing TPMS was successfully achieved in Matlab, based on the existing literature^21^. The design formulas for G-structure and D-structure porous unit structures, as illustrated in Eqs. (1) and (2), were employed to accomplish this.

1 $$\begin{aligned} \phi _{{\text{G}}} (x,y,z)7 = & \cos (x) \cdot \sin (y) + \cos (y) \cdot \sin (z) + \cos (z) \cdot \sin (x) \\ & \quad + 0.{\text{08}}[{\text{cos}}({\text{2}}x) \cdot {\text{cos(2}}y) + \cos (2y) \cdot \cos (2z) \\ & \quad + \cos (2z) \cdot \cos (2x)] + t \\ \end{aligned}$$

2 $$\begin{aligned} \phi _{{\text{D}}} (x,y,z) & = \sin (x) \cdot \sin (y) \cdot \sin (z) + \sin (x) \cdot \cos (y) \cdot \cos (z) \\ & \quad + \cos (x) \cdot \sin (y) \cdot \cos (z) + \cos (x) \cdot \cos (y) \cdot \sin (z) \\ & \quad - 0.07[\cos (4x) + \cos (4y) + \cos (4z)] + t \\ \end{aligned}$$

**Figure 1:**
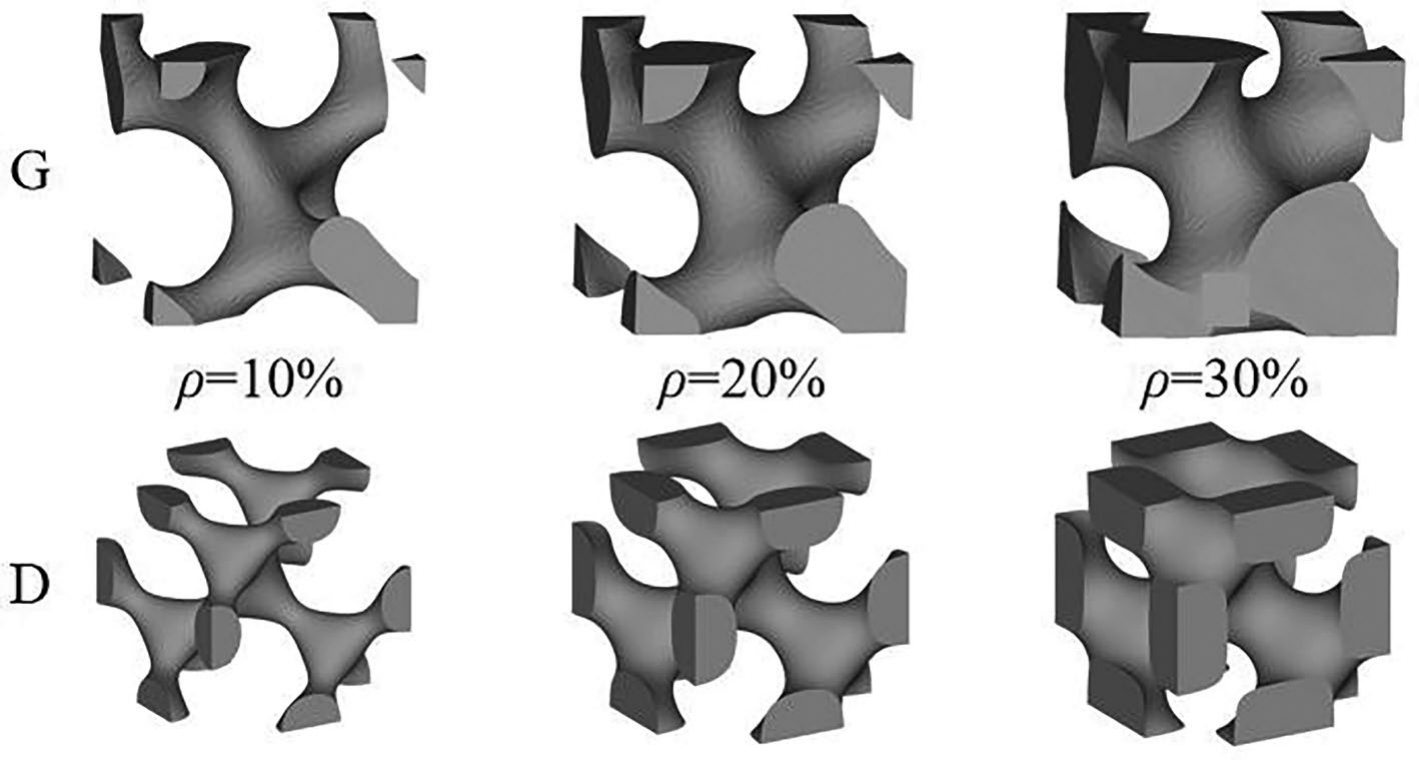
Three relative density G structures (top) and D structures (bottom) are designed.

In the formula, $\phi_{{\text{G}}} (x,y,z)$ and $\phi_{{\text{D}}} (x,y,z)$ represent the adjustable functions of G structure and D structure along the coordinate axis respectively. The variable parameter t was employed to regulate the relative density of G structure and D structure, thereby generating porous unit structures with varying volume fractions. Different values were assigned to parameter t based on the aforementioned design formula in order to create porous unit structures with diverse volume fractions. The prioritization of designing porous structures with optimal comprehensive performance for solid metal implants was undertaken. Ultimately, based on the relationship between relative density and variable parameters, t values of 0.61, 0.92, and 1.22 were selected for designing G structures, while t values of 0.49, 0.76, and 1 were chosen for D structures respectively. As shown in Fig. 1, it is a single cell structure with 10%, 20% and 30% relative density of G structure and D structure.

**Figure 2:**
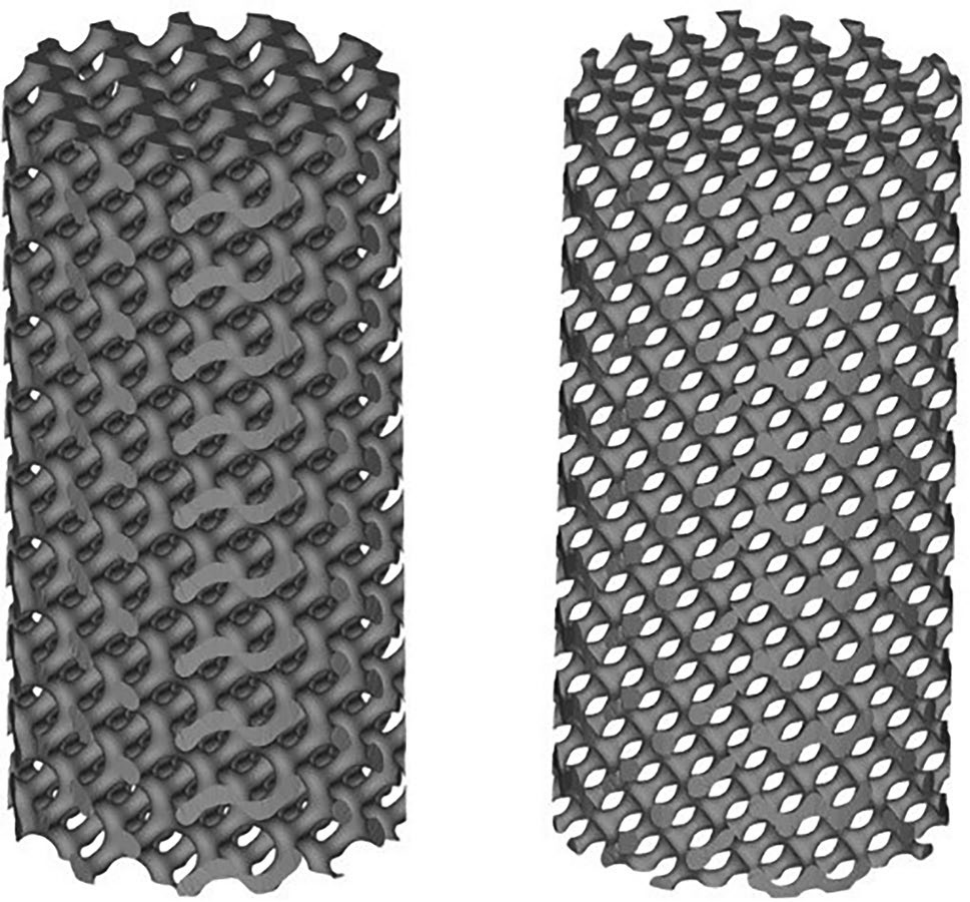
Digital design model of porous sample.

The relationship between relative density and the variable parameter was utilized for selecting designs for G structure and D structure. After designing the porous unit structure in Matlab, it was imported into Magics software in STL format to generate a digital model representing the appearance of the porous sample in Fig. 2. The cylindrical porous sample model has a height of 20 mm and a radius of 5 mm.

**Figure 3:**
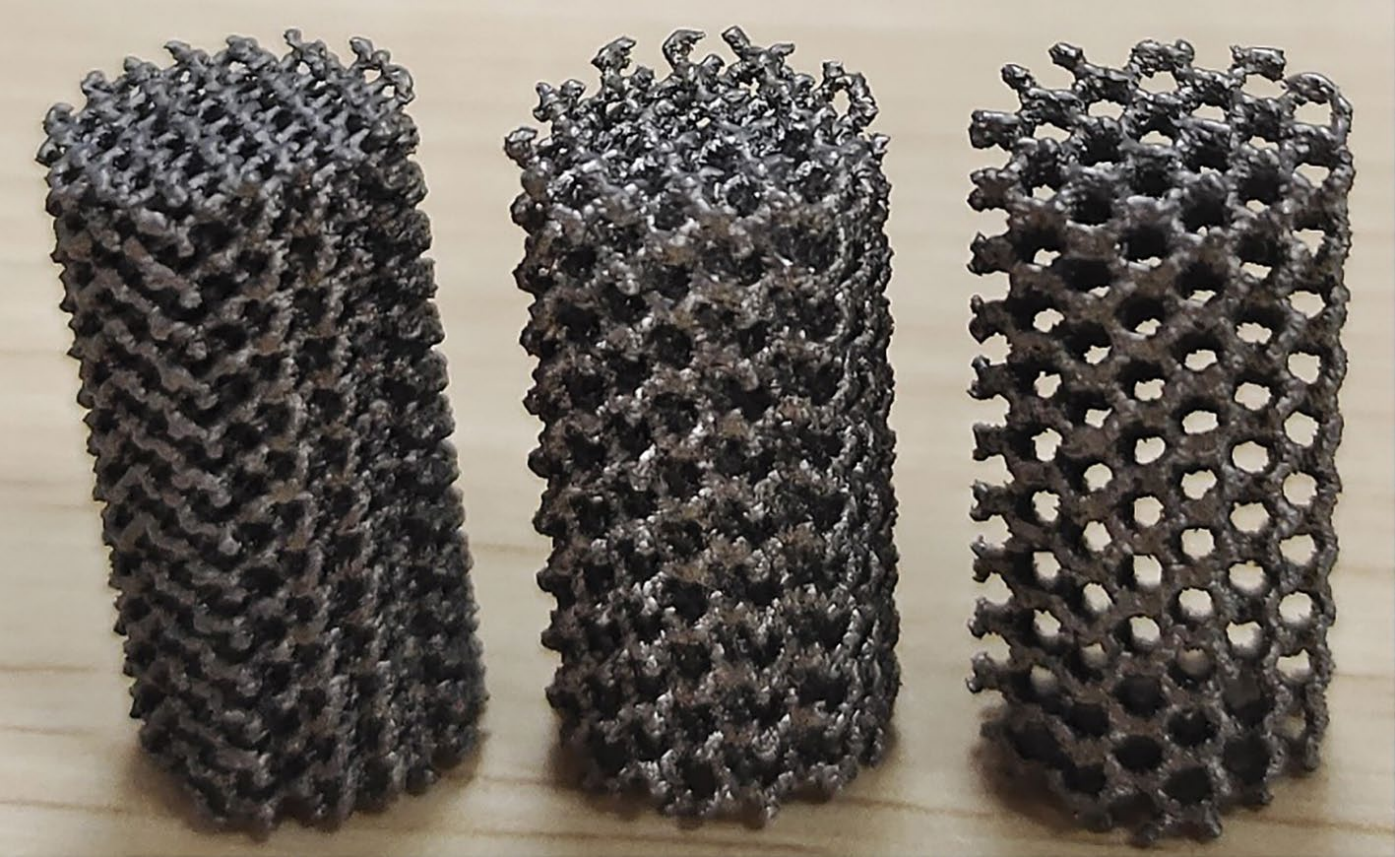
EBM printed sample (From left to right is D, $\uprho =30\text{\%}$; G, $\uprho =30\text{\%}$; G, $\uprho =20\text{\%}$).

The porous sample was prepared by EBM technology in this study. In terms of materials, Ti–6Al–4V is selected, which is an α + β type titanium alloy with high specific strength and good biocompatibility, and is the most widely used titanium alloy material in the medical field. In the range of 50–95% porosity, the elastic modulus of Ti–6Al–4V alloy porous material prepared by SEBM can reach 0.1–20.0 GPa, which is similar to the elastic modulus of bone trabecula and cortical bone. The digital model of the designed porous sample was imported into the pre-processing software provided by the EBM printer (Arcam, Sweden).The printing process employed Ti–6Al–4V medical-grade powder with a particle diameter range of 65 µm to 105 µm for titanium alloy particles. Prior to powder deposition, the substrate was preheated until it reached a temperature of 650 °C. Subsequently, the necessary electron beam parameters for printing were configured, including a current size of 30 mA, a scan line length of 45 mm, and a scanning speed of 4560 mm/s. Finally, the porous sample was fabricated through layer-by-layer stacking. The ultrasonic cleaning equipment was employed to effectively cleanse porous samples, thereby eliminating excessive impurities such as adhesive powder and surface dust. The duration of the ultrasonic cleaning process was carefully regulated to approximately 15 min, utilizing a frequency of around 40 kHz. Glass beads with a particle size measuring about 63 µm were utilized to dislodge any remaining particles from the porous samples. Subsequent microscopic examination revealed partial branch fractures within the porous samples possessing a relative density of 10% and unit size measuring 1 mm. Part of the printed sample is shown in Fig. 3.

### Compression and stress–strain tests

The mechanical test was conducted on two sets of porous specimens according to the "GB/T7314-2017—Test method for compression at room temperature of metallic materials"^22^. The experimental setup employed the TSE105D microcomputer-controlled electronic universal testing machine, manufactured by Shenzhen WANCE Testing Equipment Co., Ltd. TestPilot V2.1.1014 software was utilized for data analysis and processing. To minimize the impact of unexpected factors and ensure accurate results, three sets of repeated experiments were conducted on samples with identical porous structures for comparison and analysis. The mechanical experiment involved a uniaxial static compression test, employing cylindrical porous structures as the sample shape. A force of 50N was applied at the entrance, while maintaining a preloading speed and an average rate of 1 mm/min throughout the compression process. The triggering stop condition was met when the strain of the sample reached 60% or complete destruction occurred, ensuring consistency in each experimental plan. Photographs of the porous specimen were captured before and during the mechanical experiment.

The TestPilot software collected real-time support and displacement data from sensors during single-axis static compression tests, which were subsequently stored in a database for further analysis. Following this, the force–displacement data was processed, and stress and strain values were calculated using formulas (3) and (4).

3 $$\sigma_{{\text{S}}} = F/S = F/{\uppi }R^{2}$$

4 $$\varepsilon_{{\text{U}}} = \Delta L/H$$

In the formula, $\sigma_{{\text{S}}}$ and $\varepsilon_{{\text{U}}}$ represent stress and strain respectively; F represents the applied force (N); S is the area of force (mm^2^); R is the radius of cylindrical porous specimens (mm); δ represents the deformation of porous specimens (mm); H is the height of porous specimens (mm). Stress–strain curves for porous specimens with D and G structures were plotted using Origin2018 software (OriginLab, USA), employing stress calculation formula and strain calculation formula. Three sets of repeated experiments were conducted with varying unit sizes and relative densities to obtain average values of stress–strain curves for comparative analysis, aiming to determine the optimal structure.

### Design of porous bone graft structure

**Figure 4:**
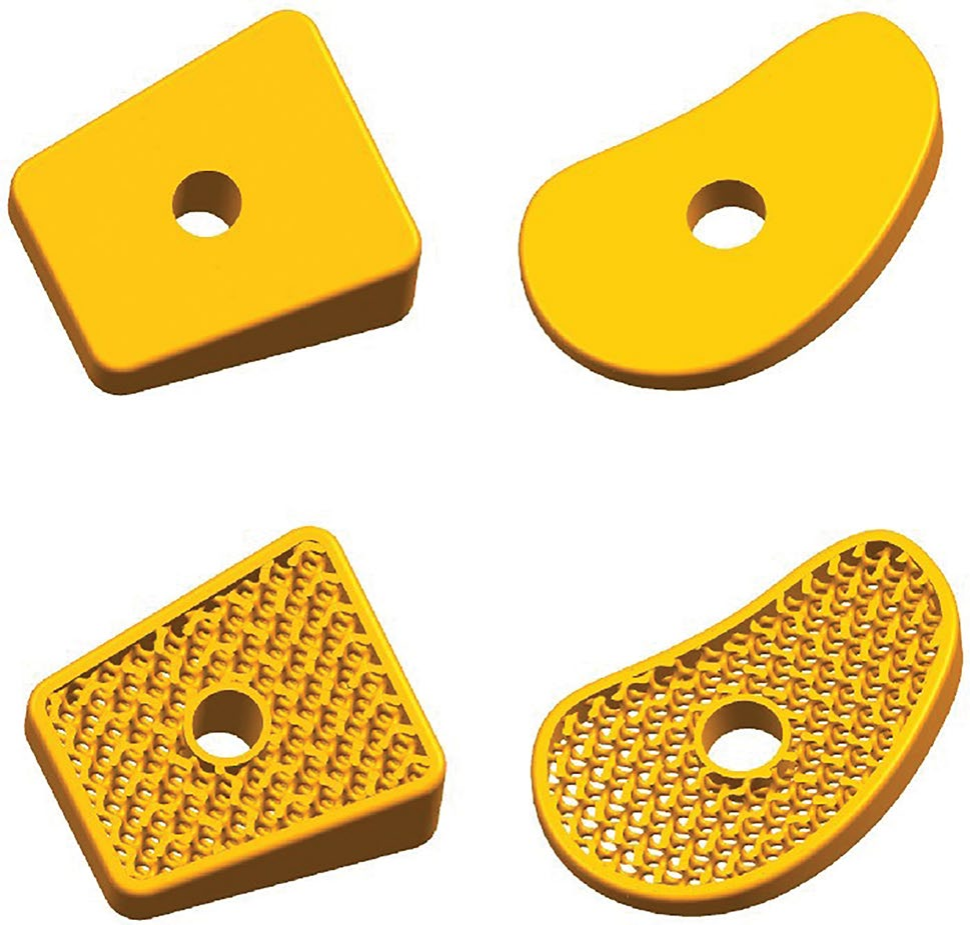
Design model of osteotomy block and porous osteotomy block.

The modeling of bone graft implants required for Evans osteotomy and Cotton osteotomy techniques in UG was achieved by referencing a model. By incorporating the specific dimensions of the calcaneus and medial cuneiform extracted from a three-dimensional reverse-engineered foot model, precise measurements were determined to customize personalized product models for patients, as illustrated in Fig. 4.

### Finite element simulation

**Figure 5:**
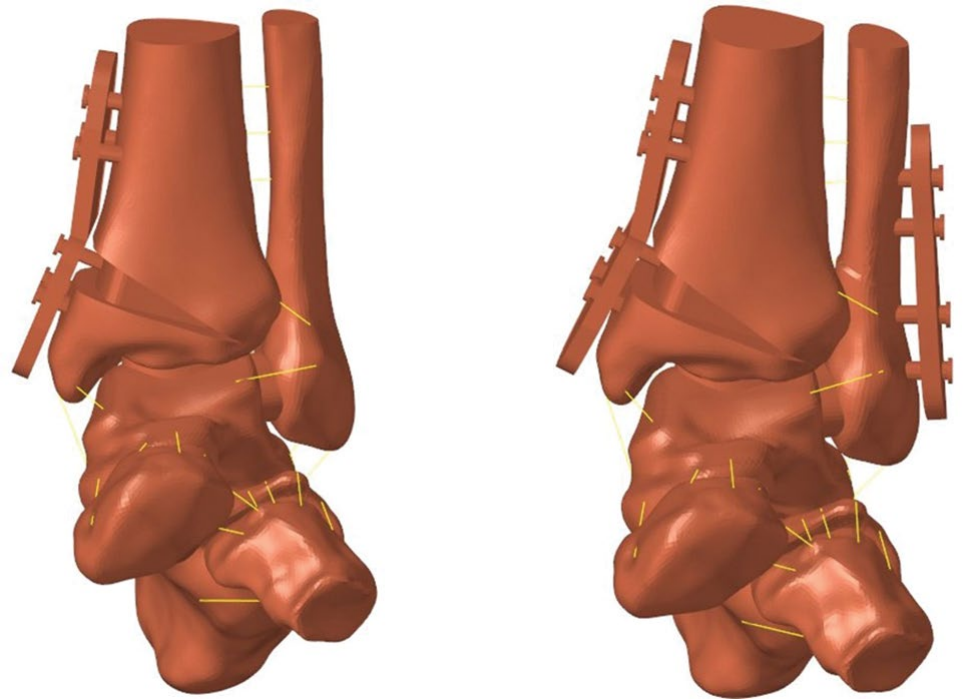
Middle and rear foot mesh model. (**a**) Preserving fibular; (**b**) Cutting fibular.

Mimics software was used to extract calcaneus, cuboid and scaphoid models again based on 2D CT image data. Then, these three bone models were imported into UG software and combined with the ankle joint, and related cartilage was constructed at the same time to complete the reconstruction of the middle and hind foot model, and the TAS, TT and TC angles of the varus middle and hind foot model were measured to facilitate the comparison after simulation. For the supralleolar osteotomy model with the fibula preserved, the osteotomy was positioned at about 4.7 cm above the medial malleolar tip in UG software, a reference plane was created for the tibial split model, rotation command was selected to correct the position of the tibia and fibula, and finally all parts were assembled into a whole. As for the supralleolar osteotomy model of the fibula amputation, the fibula was overall oblique osteotomy on the basis of retaining the fibula model, and the osteotomy gap was about 2 mm. As shown in Fig. 5, the mesh models of the preserved fibula and the truncated fibula are presented.

After the geometric solid model is established, it is respectively imported into HyperMesh software for mesh division and ligament creation. Then, the model is imported into Abaqus software in the format of INP file to start the assignment of material properties, establishment of analysis steps, contact setting, load and boundary conditions setting and other operations. In this study, the normal standing posture of both legs was simulated. The applied load size was 360 N, and the force distribution of the tibia and fibula was 5:1, that is, the applied load size of the tibia was 300 N, and the applied load size of the fibula was 60 N, and the load type was set as concentrated load. Two reference points were created on the upper surface of the tibia and fibula and coupled to make their forces uniform. The distal calcaneus was completely fixed, the scaphoid and cuboid moved freely in the X direction and were all fixed in the Y and Z directions, while the tibia and fibula moved freely in the Z direction and were all fixed in the X and Y directions. The distribution of material properties is shown in Table 1. Each contact Surface of the model was established in the Surface manager of Abaqus software. Secondly, the interaction was created in the "Mutual contact Properties" module. The normal contact between talus and cartilage was "hard contact" to simulate the relative sliding between joints. The tangential contact between cartilage and talus was set as "penalty function", the friction coefficient μ was 0.01, while the friction coefficient μ was 0.2 between osteotomy and tibia. At the same time, the slip formula in the "Interaction" module is "limited slip", and the "Specify tolerance for adjustment area" is checked. The tolerance value is 0.05, and other parameters are set to the default values of the system. The simulation solution is completed in ABAQUS software.

**Table 1:** Material properties of implant and ankle joint tissues.

Finite element model	Modulus of elasticity (MPa)	Poisson’s ratio
bone	7300	0.30
gristle	10	0.40
ligamenta	260	0.49
Bone plates, osteotomy pieces and screws	110,000	0.30

### Ethics approval and consent to participate

The study was approved by the Medical Ethics Committee of The General Hospital of Western Theater Command (20190062). All patients included in this study gave their informed consents.

## Results

### The correlation between relative density and variable parameters of porous structures

**Figure 6:**
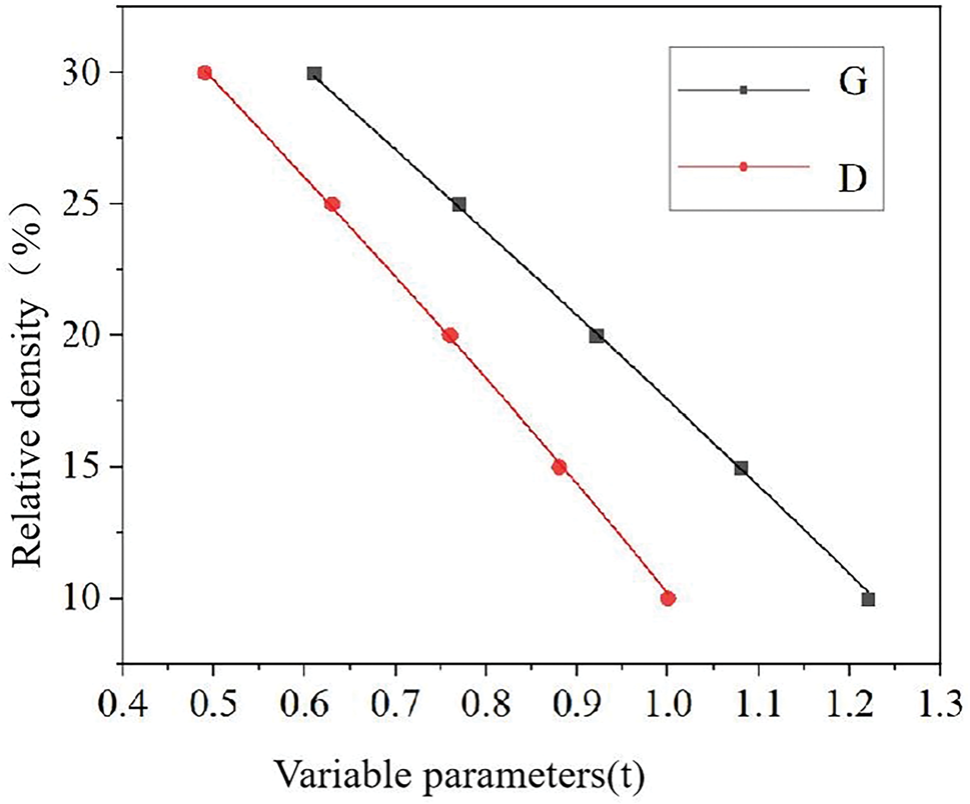
Relationship between relative density and variable parameters.

By fitting the curve, a mathematical relationship between the variable parameter t and relative density ρ was established, as illustrated in Fig. 6.

The equations for calculating the relative density of two distinct porous structures, namely G structure and D structure, were provided by Eq. (5) and (6) respectively. Based on the graph of the fitted curve, it is evident that a negative correlation existed between relative density and variable parameters. In porous structures, lower relative density corresponds to a smaller volume fraction, thereby imparting lighter characteristics when utilized for manufacturing porous implants. However, excessively low relative density may have implications for the quality of structural components during the 3D printing process.

5 $$\rho_{{\text{G}}} = - 1.82t^{3} + 2.75t^{2} - 32.45t + 49.06$$

6 $$\rho_{{\text{D}}} = - 6.1t^{3} + 8t^{2} - 40.42t + 48.69$$

### Compression test

**Figure 7:**
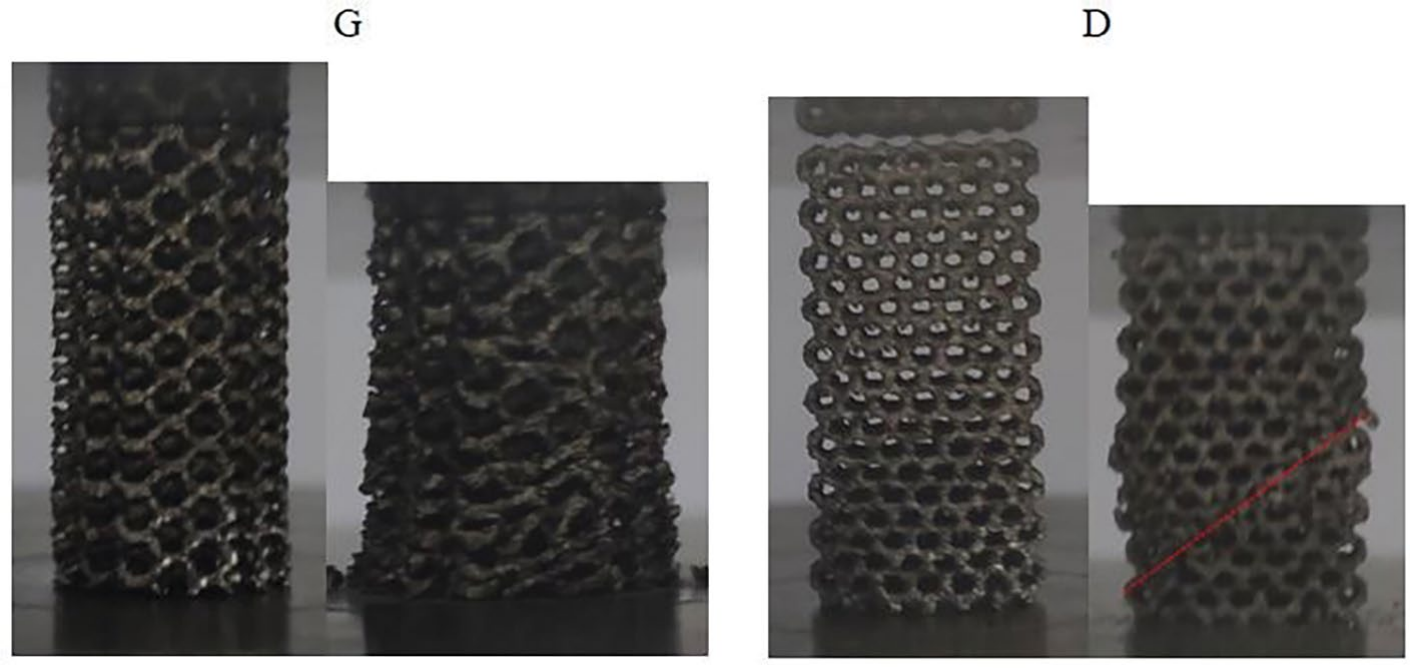
Failure form of porous sample.

The failure modes of porous specimens were analyzed, as illustrated in Fig. 7. From the compression failure images of the porous specimens, it can be observed that the G structure undergoes a progressive layer-by-layer collapse from bottom to top, while the D structure exhibited a shear failure zone at a 45° angle which impeded energy absorption and rendered it relatively more susceptible to brittle fracture compared to the G structure.

**Figure 8:**
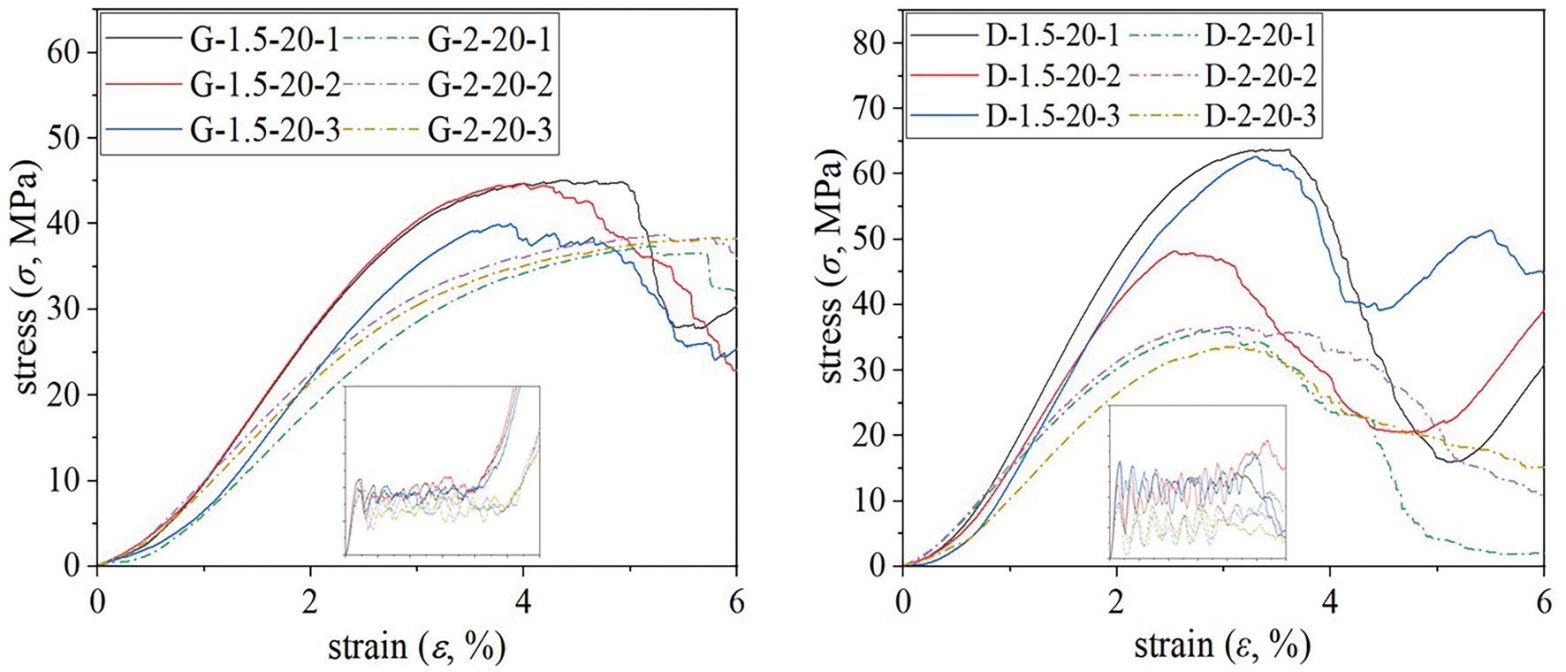
Comparison of mechanical properties of repeated tests. (**a**) G structure porous sample; (**b**) D structure porous sample.

Comparative analysis was conducted on the stress–strain curves of two types of porous structures, namely G and D structures, which possessed identical geometry but different relative densities as depicted in Fig. 8. In repeated experiments using a unit size of 2 mm, both G and D structure samples exhibited similar stress–strain curves with no statistically significant differences in mechanical strength. However, when the unit size was reduced to 1.5 mm, the repeatability of the stress–strain curve remained robust for G structure porous samples while D structure porous samples displayed greater deviations.

**Figure 9:**
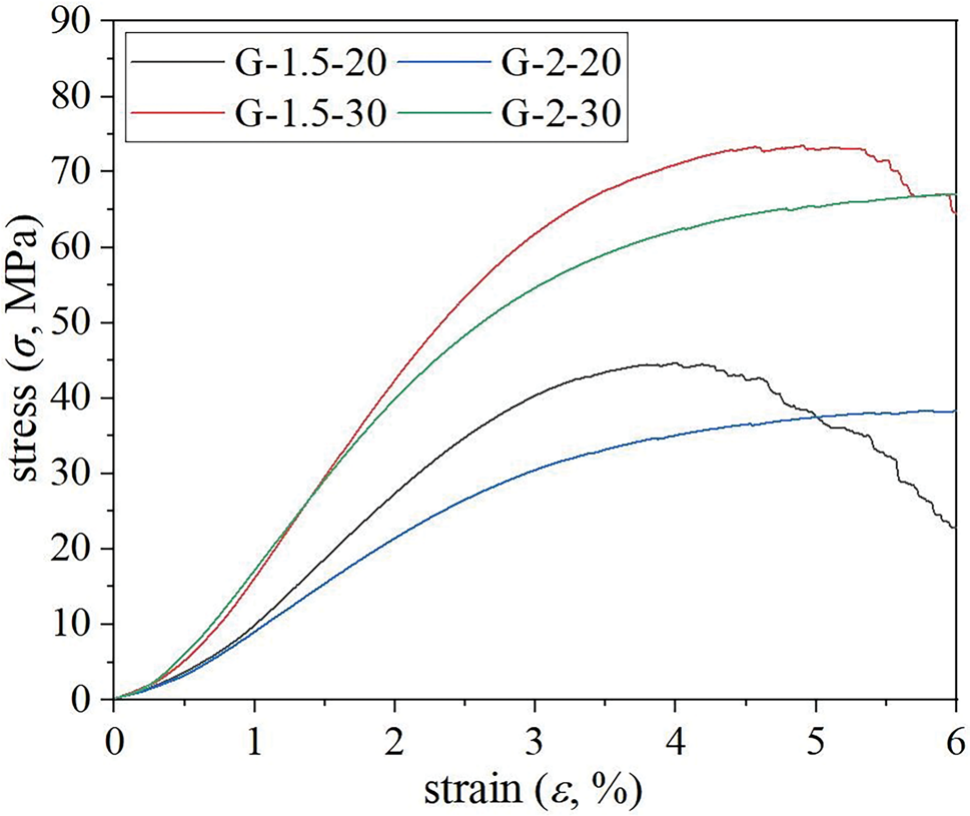
Stress–strain curve of G structure.

The optimal porous structure for designing bone graft implants was determined through an analysis of various porous samples with a G structure. A comparative analysis of stress–strain curves was conducted, considering different unit sizes and relative densities of the G structure, as depicted in Fig. 9.

The mechanical properties of porous specimens indicate that G-structured specimens with a relative density of 30% demonstrate superior mechanical strength and a higher elastic modulus for the same unit size. Conversely, porous specimens with a relative density of 20% may not possess as high mechanical strength as the former; however, they do exhibit lower elastic modulus and larger porosity. This facilitates nutrient transfer between both sides of bone tissue and enhances bone ingrowth ability while better matching the elastic modulus of adjacent skeletal units in Table 1.

### Simulation results and analysis

**Figure 10:**
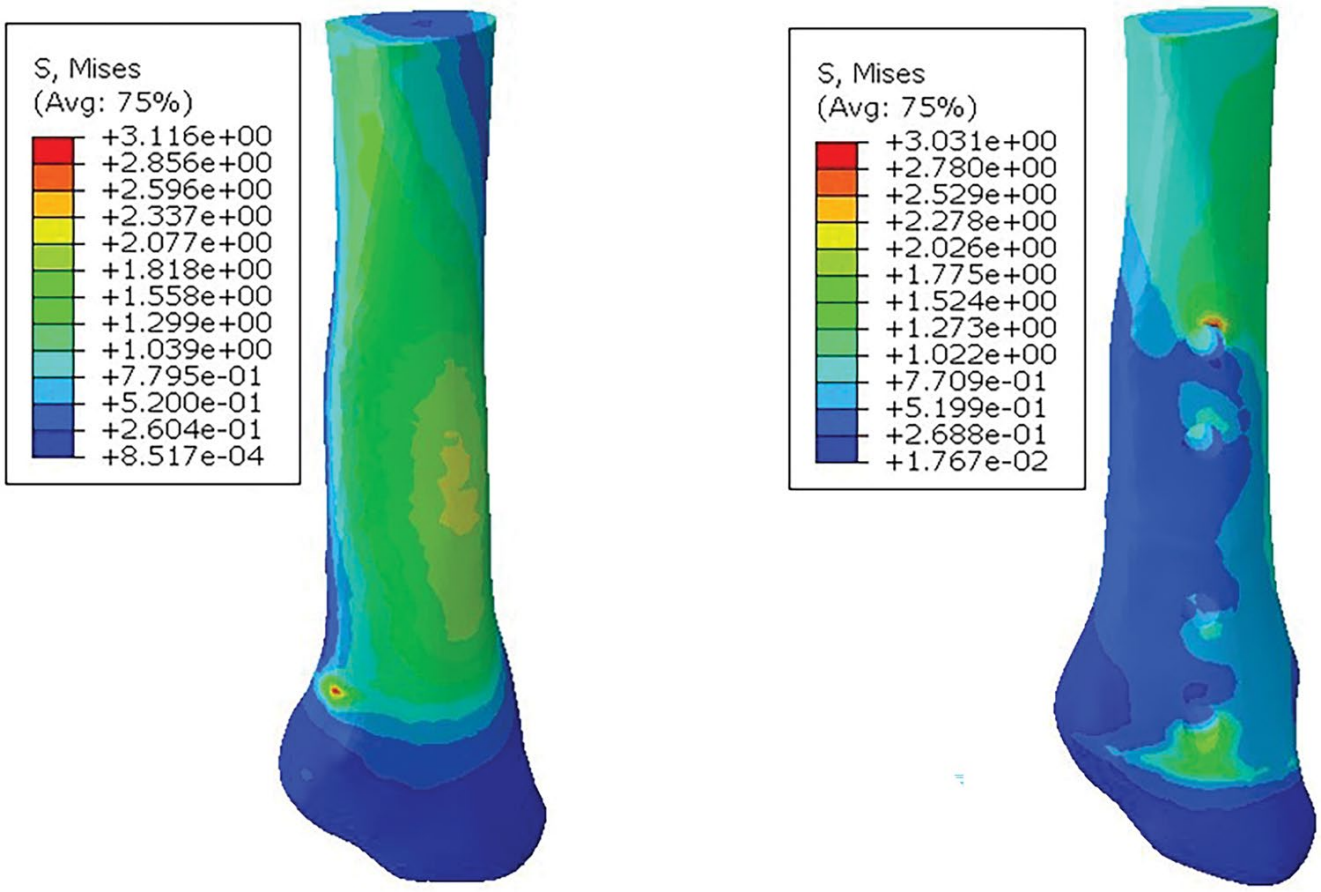
Fibular stress cloud diagram. (left) Preserving fibular; (right) Cutting fibular.

As shown in Fig. 10, the maximum surface stress of the preserved fibula was 3.116 MPa, and the stress range was 8.517e−04–3.116 MPa. The maximum surface stress of fibula in the amputated fibula group was 3.031 MPa, and the stress range was 1.767e−02–3.031 MPa. The stress distribution of the fibula was slightly different between the two groups. The stress distribution of the former was mainly in the middle part of the fibula, while the latter was mainly in the upper part of the fibula. It is worth noting that the fibula in the fibula amputation group showed stress concentration at the first locking screw hole, and the overall stress was less than that in the fibula retention group, indicating that combined fibula osteotomy can reduce the stress on the ankle joint during supralolar osteotomy. In addition, the peroneal fretting of the former was greater than that of the latter, which once again confirmed the rationality of peroneal osteotomy.

**Figure 11:**
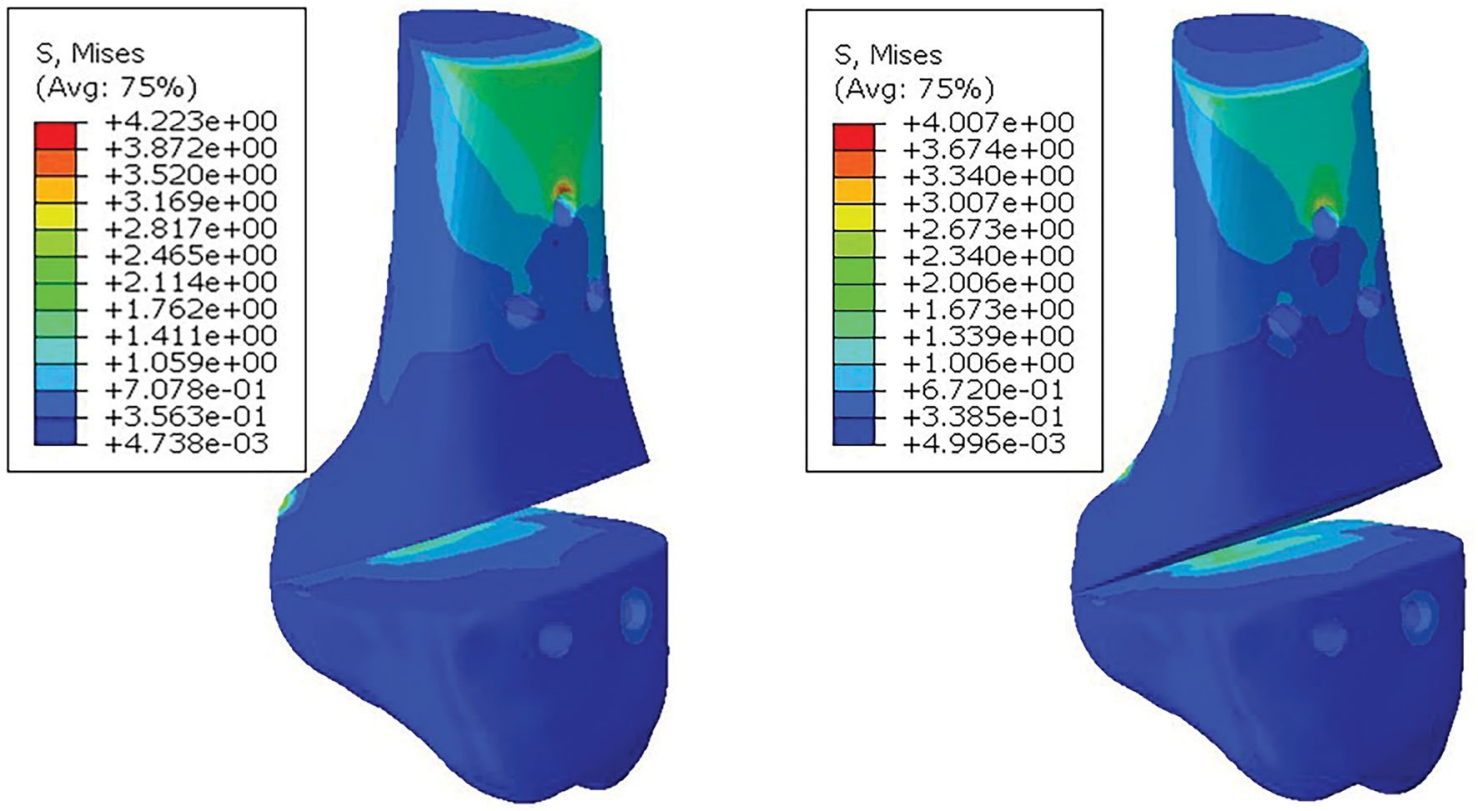
Tibia stress cloud diagram. (left) Preserving fibular; (right) Cutting fibular.

As shown in Fig. 11, the maximum surface stress of the tibia in the fibula retaining group was 4.223 MPa, and the stress range was 4.739e−03–4.223 MPa. In the fibula amputation group, the maximum surface stress of tibia was 4.007 MPa, and the stress range was 4.996e−03–4.007 MPa. In the two groups, the stress was mainly distributed in the upper end of the tibia, the lateral osteotomy site and the lower end of the tibia, in addition, the first screw hole of the fibula was partially concentrated. In addition, the maximum stress value of the tibial bone plate in the fibula retaining group was 6.040 MPa, while that in the fibula amputation group was 5.440 MPa, which proved that fibula amputation could also improve the stress situation of the bone plate.

**Figure 12:**
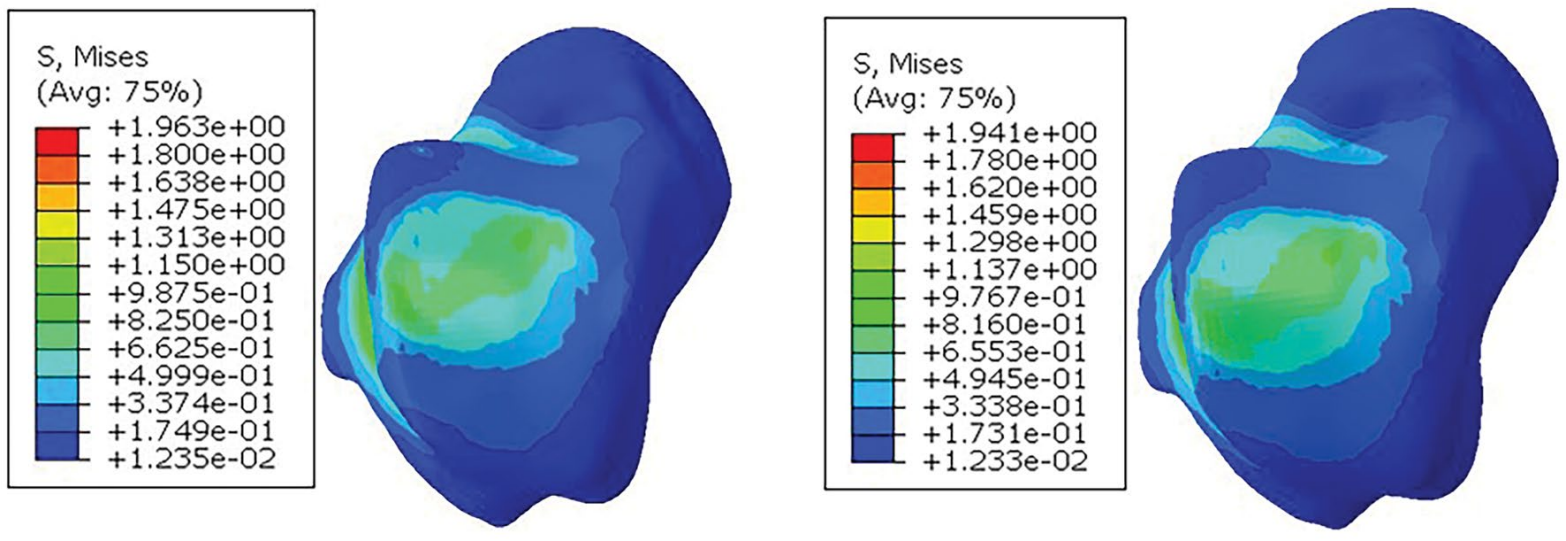
Talus stress cloud diagram. (left) Preserving fibular; (right) Cutting fibular.

As shown in Fig. 12, the maximum surface stress value of talus in the fibula retaining group was 1.963 MPa, and the stress range was 1.235e−02–1.963 MPa. The maximum surface stress of talus in fibula amputation group was 1.941 MPa, and the stress range was 1.233e−02–1.941 MPa. The results of the talus simulation experiment in the two groups were close, and the contact pressure distribution was mainly in the middle and anterolateral regions of the talus surface, which was consistent with the pressure distribution of the normal foot, indicating that whether the fibula was truncated during supraldylar osteotomy could significantly relieve the symptoms of varus ankle arthritis, or even restore the level of the normal foot.

In summary, when the elastic modulus of solid osteotomy is set to 110 GPa (titanium alloy), its maximum stress value is 4.826 MPa, which is greater than the surrounding bone tissue. After the elastic modulus of the porous structure (1332 MPa) was assigned to the osteotomy block, the maximum stress value of the osteotomy block in the fibula retaining group was 1.325 MPa, and the maximum stress value of the osteotomy block in the fibula amputing group was 1.344 MPa, which was lower than that of the nearby talus, tibia and scaphoid bone. The results show that reducing the elastic modulus of the osteotomy can indeed increase the mechanical stimulation of the bone tissue, avoid the occurrence of stress shielding phenomenon, increase the osteogenic ability of the tibial osteotomy, and contribute to the healing of the surgical wound.

## Discussion

In order to explore the changes in stress distribution between porous osteotomy implants and solid osteotomy implants when they are used for orthopedic osteotomy of flat feet, as well as the advantages of porous osteotomy blocks, porous samples with different relative densities and unit sizes of G structure and D structure were prepared by 3D printing technology, and the advantages and disadvantages of different porous structures were compared and analyzed by uniaxial static compression test. Finally, the best porous structure for porous osteotomy was determined to be G2-20 (unit size is 2 mm, relative density is 20%), and combined with similar osteotomy products for Evans osteotomy and Cotton osteotomy, a digital model of porous osteotomy for osteotomy simulation was designed. The finite element simulation results show that the elastic modulus of the porous structure is used to replace the elastic modulus of the solid osteotomy block, and the reduction of the elastic modulus of the osteotomy block can increase the mechanical stimulation of the bone tissue, avoid the occurrence of stress shielding phenomenon, and contribute to the postoperative wound healing and improve the osteogenesis ability of the osteotomy site.

The study primarily investigated the design concepts based on TPMS, provided a comprehensive elucidation of porous unit structures, and integrated Matlab software to accomplish the modeling design of porous unit structures for fabricating foot osteotomy block samples.

The utilization of 3D printing technology, with its distinctive additive manufacturing method, enables the precise fabrication of intricate and diverse orthopedic implants. The metal implant surface exhibits a porous structure characterized by interconnected periodic distribution of bone trabeculae, providing ample space for attachment, differentiation, and proliferation of bone cells^23^. The porous structure of this implant effectively mitigated its stiffness, allowing it to conform to the mechanical properties of the human body while minimizing or preventing stress shielding. Metallic implants that mimic cancellous bone structure exhibit remarkably high biological activity and play a pivotal role in facilitating osseointegration on their surfaces, thereby augmenting the long-term stability of implanted prostheses^24,25^. Consequently, the design of porous structures on 3D printed metal implant surfaces has emerged as a prominent research focus within the field of orthopedic implants^26,27^.

The TPMS exhibit a multitude of advantages, encompassing porosity, smoothness, connectivity, diversity, and controllability. These surfaces demonstrate periodicity along three independent directions in three-dimensional space with seamless continuity and a significantly high specific surface area^11,28^. The surface exhibited a diverse range of geometric shapes, and the pore size and porosity of the strut structure could be adjusted by modifying function parameters. Furthermore, these surfaces demonstrated periodic variation in space while retaining characteristics such as smoothness and complete connectivity^2,29^. The structure also featured curved channels, which greatly facilitated cell attachment and migration, thereby significantly improving the rates of cell implantation in 3D printing^18^.

The design of the porous unit structure primarily considers factors such as elastic modulus, specific surface area, and porosity with the aim of optimizing the biological performance of the porous bone graft implant and ensuring compatibility with both sides of the bone tissue. Among these factors, the TPMS elastic modulus serves as a crucial evaluation index for assessing the mechanical properties of porous structures^30^. It played a pivotal role in maintaining an equilibrium between porous bone graft implants and osseous tissue^31^. Excessive elevation of the elastic modulus can give rise to stress shielding effects, thereby hindering patient recovery and leading to complications such as osteoporosis. Conversely, an excessively low elastic modulus may compromise the mechanical strength of porous implants, resulting in premature failure. Specific surface area refers to the ratio of total surface area to total volume within a porous structure^32^. A larger specific surface area signifies enhanced adsorption capacity and facilitates an expanded contact interface for cellular growth, migration, and adhesion. The porosity (φ) of porous samples is influenced by the volume fraction, with higher volume fractions resulting in lower porosity and lower volume fractions leading to higher porosity. Replicating the anatomical structures and functions of tissues, as well as designing 3D scaffolds, remains an ongoing challenge. To address this issue, it is imperative to develop scaffolds with biomorphic surfaces that effectively enhance cell attachment, proliferation, and differentiation. "In this study, the TPMS-based scaffolds were meticulously designed using specific trigonometric equations to ensure consistent porosity and unit cell count while exhibiting varying surface curvatures. When designing a porous structure, excessively high or low porosities were found to be unsuitable for application in porous implants; instead, selecting an appropriate porosity facilitated a favorable environment for cell growth and promoted ingrowth into both sides’ cut surfaces of bones^18^.

To mitigate the impact of powder adhesion and impurities on the mechanical and biological properties of porous specimens, post-processing utilizing ultrasonic cleaning can be employed. Moreover, post-processing is indispensable for enhancing the applicability of porous implants in practical scenarios^18^. Sandblasting can effectively eliminate residual powder within cleaned porous specimens, thereby minimizing potential harm to human health and enhancing their biological performance.

Due to the increased porosity, fabricating porous specimens with smaller rod diameters under identical processing conditions becomes more challenging. Furthermore, the combination of a reduced rod diameter and higher porosity complicates the removal of residual powder through sandblasting, thereby hindering the application of this structured porous implant in human bodies^33^. Although residual powder enhances surface roughness and provides an improved environment for cell attachment to promote bone tissue growth on both sides, there is a risk of detachment of adhered powder residues that may lead to secondary infection at the bone resection site and affect postoperative recovery outcomes or even result in more severe consequences. Employing implants with increased porosity and larger pore size can augment contact with bone tissue and enhance osseointegration ability in TPMS^34^.

By analyzing the stress–strain curves of three cyclically loaded minimal surfaces, it can be observed that there is minimal disparity in mechanical strength between the G and D structures with a unit size of 2 mm. However, when reducing the unit size to 1.5 mm, significant deviation becomes apparent in the porous specimens of the D structure. This discrepancy may arise from larger porous specimens having a unit size closer to the intended value, resulting in smaller manufacturing deviations; whereas controlling manufacturing accuracy for smaller porous specimens becomes more challenging^35^. The compressive strength of the D scaffolds was found to be superior in scaffolds with lower porosity, while a decrease in mechanical properties was observed as the porosity increased^28^. Conversely, G scaffolds maintain their strength even as the porosity increases. By analyzing stress–strain curves for G and D structures with a unit size of 2 mm, it can be reasonably inferred that G structure samples exhibit superior mechanical stability compared to D structure samples.

Based on the stress–strain curve and mechanical performance data table of the three-period minimum surface, it can be observed that G-structure porous specimens exhibit a certain degree of improvement as the unit size decreases under the same relative density. An inverse relationship between the mechanical properties of G-structure and unit size is evident. Among specimens with identical unit sizes, those with a relative density of 30% demonstrate higher mechanical strength and elastic modulus. However, despite the lack of comparable mechanical strength to their counterparts, porous specimens with a relative density of 20% exhibited lower elastic modulus and higher porosity, which conferred advantages in facilitating bone tissue nutrient transfer and enhancing bone ingrowth ability^2,12^. Additionally, it should exhibit better compatibility with adjacent skeletal units in terms of elastic modulus. Therefore, when meeting equivalent strength requirements, the selection of a G2-20 structure with a relative density of 20% is considered optimal for porous design purposes.

The presence of excessive sharp edges in the pure porous structure hinders implantation and patient comfort^36^, while also increasing susceptibility to failure and fracture. Therefore, it is essential to retain the solid component in order to provide structural support. The region requiring porosity can be determined through commands such as offsetting and splitting, and then exported in STL format. The identified optimal porous structure is added to the Magics database. After importing the solid structural model that requires porosification, utilize the structure design command on the main page to appropriately select and size the added porous structure for introducing porosity into the solid structure along X, Y, and Z axes. Finally, perform Boolean operations between the porous section model and solid frame section in UG software to obtain a final osteotomy block implant with desired porosity for Evans osteotomy and Cotton osteotomy.

## Conclusions

The osteotomy block was designed based on a three-period minimal surface (TPMS), and the resulting structure was fabricated using 3D printing technology. The mechanical properties of various structures were evaluated through both mechanical testing and finite element simulation. During mechanical testing, it was observed that the Gyroid structure exhibited a progressive failure mechanism from bottom to top, whereas the Diamond structure displayed a shear failure zone at a 45°angle which hindered energy absorption and increased susceptibility to brittle fracture compared to the Gyroid structure. Therefore, further research into porous osteotomy should focus on utilizing the Gyroid structure.

## Data Availability

The datasets supporting the conclusions of this article are included within the article.

## Abbreviations

TPMS: Triply periodic minimal surfaces
EBM: Electron beam melting
3D: Three-dimensional
STL: Standard template library
